# Intraoperative Macula Protection by Perfluorocarbon Liquid for the Metallic Intraocular Foreign Body Removal during 23-Gauge Vitrectomy

**DOI:** 10.1155/2017/6232151

**Published:** 2017-05-02

**Authors:** Robert Rejdak, Tomasz Choragiewicz, Joanna Moneta-Wielgos, Dominika Wrzesinska, Dorota Borowicz, Matteo Forlini, Anselm G. Jünemann, Katarzyna Nowomiejska

**Affiliations:** ^1^Department of General Ophthalmology, Medical University of Lublin, Ulica Chmielna 1, 20-079 Lublin, Poland; ^2^Department of Didactics and Medical Simulation, Human Anatomy Chair, Medical University of Lublin, Ulica Jaczewskiego 4, 20-090 Lublin, Poland; ^3^Institute of Ophthalmology, University of Modena and Reggio Emilia, Modena, Italy; ^4^Department of Ophthalmology, University Eye Hospital, Rostock, Germany

## Abstract

*Purpose.* To evaluate visual and safety outcomes of 23-gauge (G) pars plana vitrectomy (PPV) with application of perfluorocarbon liquid (PFCL) for intraoperative protection of the macula during intraocular foreign body (IOFB) removal. *Methods.* Retrospective study of 42 patients who underwent 23 G PPV for IOFB removal from posterior segment with intraoperative PFCL application for the macula shielding. Collected data included corrected distance visual acuity (CDVA), size of IOFB, and complication rate. The mean follow-up period was 12 months. *Results*. The mean preoperative CDVA was 0.54 logMAR (SD 0.46), and the final mean CDVA was 0.68 logMAR (SD 0.66). All IOFBs were metallic with mean dimensions of 4.6 mm × 2.1 mm. Twenty-two IOFBs were removed through the corneal tunnel and 20 IOFBs through the sclerotomy. No intraoperative iatrogenic lesion of the macula was observed. As a tamponade, silicon oil was applied in 31 eyes, SF_6_ gas in 5 eyes, air in 4 eyes, and 2 eyes required no tamponade. Secondary retinal detachment was observed in 17% of cases, but at the end of the follow-up, all the retinas were attached. *Conclusion*. PFCL application during PPV is a safe method of protecting the macula from unexpected falling of the metallic IOFB during its removal.

## 1. Introduction

Open globe injuries remain the most severe eye trauma. The annual incidence of open globe injuries is estimated from 3 to 7 per 100,000 per year [1, 2]. Depending on the population, open globe injuries are complicated with the presence of intraocular foreign body (IOFB) in 10 to 41% of cases. This type of injury is one of the major causes of visual impairment in a group of young males in their productive age. Most frequently, open globe injuries are work-related and occur in mechanism of hammering [3–5]. Visual prognosis depends on the localization of the entry of IOFB and damaged structures of the eye. Timing of IOFB removal is important with regard to the risk of endophthalmitis and siderosis and is a matter of discussion [6–9], whereas improper surgical technique can result in iatrogenic lesion and increased need for secondary operation.

Removal of IOFB is one of the most challenging surgery in ophthalmology. The objectives of this procedure are to remove IOFB from the eyeball in minimally invasive way and to treat lesions caused by trajectory of IOFB. In the past, all the magnetic IOFBs were extracted from the eye with an external magnet [10]. This method allowed for easy but unfortunately uncontrolled removal of the IOFB, resulting in high risk of retinal detachment (RD), caused by iatrogenic retinal breaks, produced by uncontrolled pulling of IOFB embedded in the retina or in the vitreous bands. This procedure was also associated with a high risk of vitreous haemorrhage and proliferative vitreoretinopathy (PVR) [11].

Pars plana vitrectomy (PPV) is currently a standard procedure in the treatment of pathologies located in the posterior segment of the eye. In case of IOFB, PPV allows for good visualization of IOFB, precise inspection of retinal lesions, and laser treatment of retinal breaks [7]. However, during PPV approach, IOFBs are more frequently unintentionally dropped on the macula compared to removal through a sclerotomy with the external electromagnet [12].

Perfluorocarbon liquid (PFCL) was introduced firstly to vitreoretinal surgery by Stanley Chang as a temporary tamponade [13]. It has been shown that PFCL has no ability to dampen the impact force of falling IOFB measured on a force transducer [14]. A more recent study has shown experimentally that PFCL has the facility to shield the macula from the impact of dropped metallic IOFB by deflecting its trajectory on the PFCL-balanced salt solution (BSS) interface [15].

The aim of this study was to analyse the clinical results of 23-gauge (G) PPV with intraoperative application of PFCL to protect the macula from unexpected falling of metallic IOFB during its removal.

## 2. Materials and Methods

Data were collected retrospectively from medical reports of 42 consecutive patients treated at the Department of General Ophthalmology, Medical University of Lublin, Poland, between August 2009 and November 2015. The treatment chosen in the study was a part of a standard care. All patients were routinely fully informed about the risk and benefits of the surgery and the written consent was obtained. The study was performed in accordance with the Declaration of Helsinki.

Pre-, intra-, and postoperative data were collected. Preoperative data included demographic data, corrected distance visual acuity (CDVA) measured with Snellen's decimal scale converted to logMAR, intraocular pressure (IOP), pre-existing pathology, entry site of the IOFB, lesion of eye structures, history of the disease, and time interval between accident and operation. For CDVA analysis, finger counting and hand movement were calculated in decimal values [16]. Presence of IOFB in the posterior segment of the eye was confirmed by preoperative computed tomography (CT) examination of the orbit and eyeball. Intraoperative data included localization of site of IOFB removal, course of the operation, type of intraocular tamponade, and intraoperative complications.

Postoperative data from follow-up visits included CDVA, slit lamp findings, and IOP. The schedule of postoperative examinations was as follows: on the first day, one week, one month, and 4, 6, 9, and 12 months after surgery.

Dimensions of metallic IOFB were measured with a micrometre.

### 2.1. Surgical Technique

**Figure 1:**
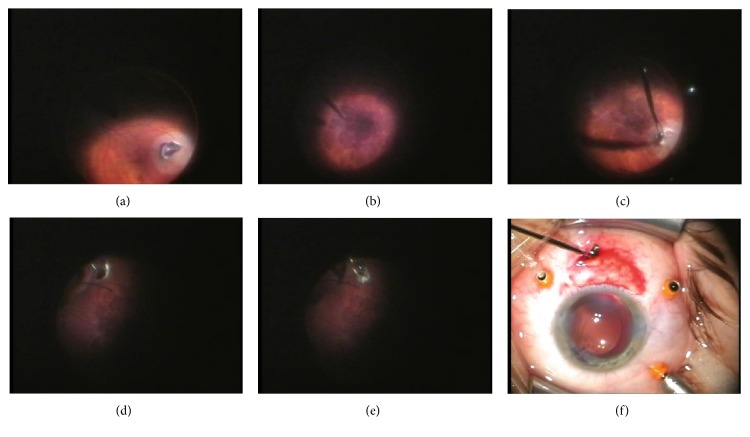
(a) Intraretinal localization of IOFB, (b) application of PFCL on the macula, (c) grabbing IOFB and attempt of its removal, (d) free-floating IOFB on the surface of PFCL after its unintentional fall, (e) grasping IOFB from PLCL surface, and (f) IOFB removal through sclerotomy.

**Figure 2:**
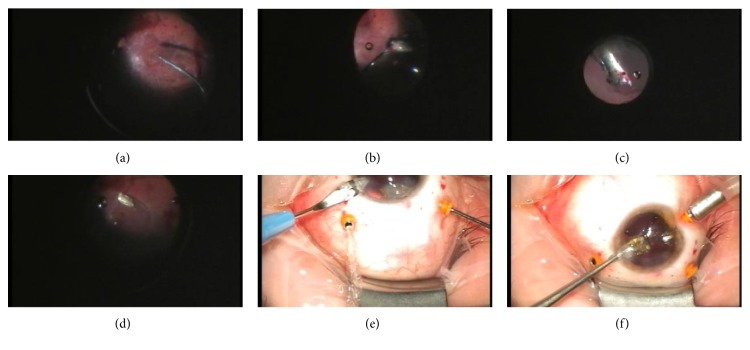
(a) Application of PFCL on the macula, (b) grabbing IOFB, (c) elevating IOFB toward the anterior chamber, (d) deflection of its trajectory by PBS-PFCL interface from the macula toward the peripheral retina during unintentional IOFB fall, (e) clear corneal incision enlargement, and (f) IOFB removal through corneal tunnel incision.

All surgeries were performed under general anaesthesia by experienced vitreoretinal surgeons. All patients were treated with 23 G PPV (Constellation, Alcon, Fourth Worth, USA). First, the site of corneal entry of IOFB was sutured with Nylon 10.0 sutures and the sclera with Vicryl 7.0 sutures. Crystalline lens considered to be opaque or injured was removed with phacoemulsification or cutter, and when possible, foldable, acrylic intraocular lens (IOL) was implanted to the capsule or the sulcus during the primary surgery. The posterior hyaloid detachment was induced in each patient with assistance of triamcynolone. After performing complete vitrectomy with indentation in all cases, intraoperative PFCL (F-Decalin, Fluoron GmbH, Ulm, Germany) was applied to protect the macula during IOFB removal. The PFCL was applied in the amount of 1 ml to cover the posterior pole to arcades including the macula. IOFB was removed through sclerotomy (Figure 1) or clear corneal tunnel incision (Figure 2). All retinal breaks were treated with laser endophotocoagulation. At the end of the operation, PFCL was removed completely. If intraocular tamponade was necessary, air, 25% sulfur hexafluoride (SF_6_) gas, or 5000 Cst silicone oil was used. In case of primary macula laceration, internal limiting membrane (ILM) was peeled with assistance of indocyanine green (ICG). Vancomycin dissolved in the infusion fluid in concentration of 0.2 mg/ml suggested by Rejdak et al. [17] was used intraoperatively as endophthalmitis prophylaxis. In postoperative period, all cases received locally steroids and fluoroquinolones.

### 2.2. Statistical Analysis

For statistical analysis, Mann-Whitney test to compare two groups and one-way ANOVA test for more than two groups were applied. Pearson's correlation coefficient test was applied to measure the association between two variables (GraphPad Software Inc., La Jolla, USA). Differences were considered statistically significant at the level of *p* < 0.05.

## 3. Results

All cases were men with the mean age of 45 years (min. 15 years, max. 76 years, median 46 years, and SD 15 years). All IOFBs were metallic with mean dimensions of 4.6 mm × 2.1 mm (min. 1.6 × 1.1 mm, max. 9.5 mm × 6.8 mm, median 3.3 mm × 1.8 mm, and SD 3.3 × 1.4). Preoperative mean CDVA was 0.54 logMAR (min. no light perception, max. 0, median 1.52, and SD 0.46). Entry wound was localized in the cornea in 23 eyes and within the sclera in 19 eyes. Preoperative laceration of the retina was diagnosed in 20 eyes. Unintended IOFB fall onto PFCL was reported in 3 eyes (7%), but no eye revealed intraoperative iatrogenic lesion of the macula induced by dropped IOFB. Mean time interval between trauma and operation was 48 days (min. 0 days, max 900 days, median 1.5 days, and SD 178 days). All IOFBs were removed with 23 G forceps. Twenty-two IOFBs were removed through clear corneal tunnel incision and 20 through sclerotomy. Crystalline lens was removed in 39 eyes; IOL was implemented during primary surgery in 20 eyes and during secondary operation in 13 eyes. At the end of follow-up, 6 eyes remained aphakic. As an intraocular tamponade, silicon oil was applied in 31 eyes, SF_6_ in 5 eyes, air in 4 eyes, and 2 eyes required no tamponade. Mean observation time was 11.7 months (min. 1 month, max. 47 months, median 11 months, and SD 10.5 months). At the end of the follow-up, mean CDVA was 0.68 logMAR (min. no light perception, max 0.04, median 0.82, and SD 0.66). Compared to preoperative results, it improved in 18 (42.9%) cases, was equal in 9 (21.4%) cases, and decelerated in 15 (35.7%) eyes. Final CDVA better than 0.3 logMAR was observed in 6 (14.3%) eyes. All 3 eyes (7.1%) with no light perception at the initial visit had no light perception at the end of follow-up. No statistical significant differences of final CDVA between the eyes without lens removal, primary implantation, secondary IOL implantation and the aphakic eyes were observed (*p* = 0.72), as well as no differences between scleral and corneal entry wound localizations (*p* = 0.92) and between scleral and clear corneal localizations of the IOFB removal site (*p* = 0.92) or between air, SF_6_, silicon oil, and no tamponade (*p* = 0.96). Moderate downhill correlation between maximal dimension of IOFB and final CDVA was found (*r* = −0.33).

At the end of follow-up, mean IOP was 15.6 mmHg (min. 3 mmHg, max. 30 mmHg, median 16 mmHg, and SD 5.3 mmHg). Increased IOP was controlled with topical antiglucomatous medications in 12 eyes filled with silicon as a tamponade. Seven (16.6%) eyes required reoperation due to secondary RD. At the end of follow-up, the retina was attached in all the eyes. No eye had signs of endophthalmitis and atrophy and no eye required enucleation.

## 4. Discussion

In the present study, we have reported for the first time that the application of PFCL is the safe method for shielding the macula from falling metallic IOFB during PPV. We have not observed any iatrogenic injury of the macula during PPV for metallic IOFB removal with PFCL use in our group of 42 patients. However, falling of IOFB was reported in 3 patients without retinal injury within the macula.

PFCL is optically clear and has a specific gravity greater than that of water and high interfacial tension in water. High specific gravity enables removal of subretinal fluid by primary retinal breaks, eliminating the need for a retinotomy in RD. PFCL flattens the retina and mechanically fixates it, reducing the tractional forces. PFCLs are applied intraoperatively during vitrectomy for complicated RD, PVD, giant tears, and trauma [13]. Because PFCL has direct toxic effect for retinal pigment epithelium cells and induces mechanical damage of retinal ganglion cells, all PFCL should be removed from the eye at the end of surgery [18]. PFCL has been used already to protect the macula while removing luxated lens material or IOL [19]. The dislocated lenses or IOLs float in PFCL not only by its high gravity but also mostly by its high-PFCL-BSS interfacial tension, which is responsible for deflecting dropped IOFB trajectory along the interface toward the peripheral retina and thus protecting the posterior segment of the eye.

The macula can be a shield from IOFB drop during its extraction by reduction of impact force of falling IOFB or by deflecting its trajectory toward the peripheral retina. Ernst and coworkers examined the ability of different substances to dampen the impact force of an IOFB dropped inside the model of the eye and demonstrated that it could be achieved with silicone oil and viscoelastic, whereas PFCL accelerate IOFB falling compared with BSS. It is explained with lower viscosity of PFCL, what allows falling IOFB to reach a higher velocity [14]. This finding is not in contrary to the observation that the high interfacial tension of PFCL in BSS allows to deflect trajectory of dropped IOFB on the PFCL-BSS interface which was shown experimentally by Shah and colleagues [15]. These properties could be utilized only when a small bubble of PFCL is applied; otherwise, IOFB would accelerate and cause greater damage. It was proven experimentally by Shah and colleagues that the larger PFCL bubble, the higher the final speed of IFOB [15]. In the presented study, we used 1 ml of PFCL, which was sufficient to form a bubble that covered the macula with vascular arcades. This amount of PFCL was small enough not to accelerate falling IOFB. The correlation between size and shape of IOFB was shown: the protective barrier of PFCL-BSS interface could be broken by sharp and heavy IOFB [15]. In our clinical practice, we observed that even unexpected falling of IOFB does not cause lesion of the macula, as PFCL protects the posterior pole.

Nowadays, vitreoretinal surgery allows managing of the posterior segment injuries associated with IOFBs successfully. Visual outcome of PPV for IOFB removal is associated with posterior localization of the lesion and correlates with the state of macula [4, 5]. Posterior segment can be injured primary or secondary in mechanism of iatrogenic lesion caused by a falling IOFB during its extraction, which can damage the macula and optic disc and cause retinal breaks and haemorrhages and increase the risk of macular pucker. Despite recent surgical advances, RD remains the most devastating complication after ocular injury with IOFB [9, 20, 21].

In our study, we found that the rate of secondary RD is 17%. In older studies, performing PPV decreased the risk of RD in the period following IOFB extraction from 79% to 11–23% [18, 19]. The presence of secondary RD could be explained by PVR development or not correctly treated or overlooked retinal breaks. PVR can cause a traction tear or reopen treated retinal break resulting in a late onset rhegmatogenous RD. PVR development is stimulated by retinal penetration of IOFB resulting from its primary localization or by iatrogenic breaks caused by uncontrolled manipulations during its extraction [22].

Our study has some limitations: retrospective character and relatively small subgroup sample size. There is no control group as we perform routinely PPV with PFCL for IOFB removal.

## 5. Conclusion

Application of PFCL seems to be a safe and affordable method of macular protection during metallic IOFB removal during PPV. To our knowledge, it is the first clinical case series confirming physical properties of BSS-PFCL interface which deflects trajectory of falling IOFB.

## References

[1] Schmidt G. W., Broman A. T., Hindman H. B., Grant M. P. (2008). Vision survival after open globe injury predicted by classification and regression tree analysis. *Ophthalmolology*.

[2] Larque-Daza A. B., Peralta-Calvo J., Lopez-Andrade J. (2010). Epidemiology of open-globe trauma in the southeast of Spain. *European Journal of Ophthalmology*.

[3] Yeh S., Colyer M. H., Weichel E. D. (2008). Current trends in the management of intraocular foreign bodies. *Current Opinions in Ophthalmology*.

[4] Zhang Y., Zhang M., Jiang C., Qiu H. Y. (2011). Intraocular foreign bodies in China: clinical characteristics, prognostic factors, and visual outcomes in 1421 eyes. *American Journal of Ophthalmology*.

[5] Kuhn F., Mester V., Morris R. (2002). Intraocular foreign bodies. *Ocular Trauma: Principles and Practice*.

[6] Thompson J. T., Parver L. M., Enger C. L., Mieler W. F., Liggett P. E. (1993). Infectious endophthalmitis after penetrating injuries with retained intraocular foreign bodies. National Eye Trauma System. *Ophthalmology*.

[7] Parke D. W., Flynn H. W., Fisher Y. L. (2013). Management of intraocular foreign bodies: a clinical flight plan. *Canadian Journal of Ophthalmology*.

[8] Akesbi J., Adam R., Rodallec T. (2011). Intraocular foreign bodies (IOFB) of the posterior segment: retrospective analysis and management of 57 cases. *French Journal of Ophthalmology*.

[9] Chiquet C., Zech J., Denis P., Adelin P., Trepsat C. (1999). Intraocular foreign bodies. Factors influencing final visual outcome. *Acta Ophthalmolica Scandinavica*.

[10] Mittra R. A., Mieler W. F. (1999). Controversies in the management of open-globe injuries involving the posterior segment. *Survey of Ophthalmology*.

[11] Mester V., Kuhn F. (1998). Ferrous intraocular foreign bodies retained in the posterior segment: management, options and results. *International Ophthalmology*.

[12] Wickham L., Xing W., Bunce C., Sullivan P. (2006). Outcomes of surgery for posterior segment intraocular foreign bodies—a retrospective review of 17 years of clinical experience. *Graefe’s Archive for Clinical and Experimental Ophthalmology*.

[13] Chang S., Ozmert E., Zimmerman N. J. (1988). Intraoperative perfluorocarbon liquids in the management of proliferative vitreoretinopathy. *American Journal of Ophthalmology*.

[14] Ernst B. J., Velez-Montoya R., Kujundzic D., Kujundzic E., Olson J. L. (2013). Experimental measure of retinal impact force resulting from intraocular foreign body dropped onto retina through media of differing viscosity. *Clinical and Experimental Ophthalmology*.

[15] Shah C. M., Gentile R. C., Mehta M. C. (2016). Perfluorocarbon liquids’ ability to protect the macula from intraocular dropping of the metallic foreign bodies: a model eye study. *Retina*.

[16] Händel A., Jünemann A., Prokosch H. U. (2009). Webbasierteelektronische Krankenakteim Rahmeneinesintegrierten Versorgungskonzepts als Instrument der Qualitätssicherung. *Klinische Monatsblätter für Augenheilkunde*.

[17] Rejdak R., Choragiewicz T., Kalinowska A. (2016). Vancomycin in infusion during vitrectomy in surgical treatment of acute postoperative and posttraumatic endophthalmitis. *BMC Infectious Diseases*.

[18] Inoue M., Iriyama A., Kadonosono K., Tamaki Y., Yanagi Y. (2009). Effects of perfluorocarbon liquids and silicone oil on human retinal pigment epithelial cells and retinal ganglion cells. *Retina*.

[19] Liu K. R., Peyman G. A., Chen M. S., Chang K. B. (1991). Use of high-density vitreous substitutes in the removal of posteriorly dislocated lenses or intraocular lenses. *Ophthalmic Surgery*.

[20] Soheilian M., Feghi M., Yazdani S. (2005). Surgical management of nonmetallic and nonmagnetic metallic intraocular foreign bodies. *Ophthalmic Surgery Lasers and Imaging*.

[21] Wani V. B., Ai-azmi M., Thalib L. (2003). Vitrectomy for posterior segment intraocular foreign bodies: visual results and prognostic factors. *Retina*.

[22] Pavlovic S., Schmidt K. G., Tomic Z., Dzinic M. (1998). Management of intraocular foreign bodies impacting or embedded in retina. *Australian and New Zealand Journal of Ophthalmology*.

